# An In Vivo Observational Histological Study of Peripheral Arterial Damage in Patients with Acute Limb Ischemia in SARS-CoV-2 Infection

**DOI:** 10.3390/diagnostics12020488

**Published:** 2022-02-14

**Authors:** Sorin Barac, Roxana Ramona Onofrei, Codruta Lazureanu, Robert Barna, Adrian Tutelca, Andreea Luciana Rata

**Affiliations:** 1 Department of Vascular Surgery, Research Center in Vascular and Endovascular Surgery, “Victor Babes” University of Medicine and Pharmacy, 300041 Timisoara, Romania; sorinbarac@gmail.com (S.B.); rataandreealuciana@gmail.com (A.L.R.); 2 Department of Rehabilitation, Physical Medicine and Rheumatology, Research Center for Assessment of Human Motion, Functionality and Disability, “Victor Babes” University of Medicine and Pharmacy, 300041 Timisoara, Romania; 3 Department of Morphopathology, “Victor Babes” University of Medicine and Pharmacy, 300041 Timisoara, Romania; dordecodru@yahoo.com; 4 Department of Internal Medicine II—Discipline of Gastroenterology and Hepatology, Discipline of Morphopathology, “Victor Babes” University of Medicine and Pharmacy, 300041 Timisoara, Romania; barna.robert27@gmail.com; 5 Department of Radiology, “Victor Babes” University of Medicine and Pharmacy, 300041 Timisoara, Romania; atutelca@gmail.com

**Keywords:** SARS-CoV-2 infection, acute limb ischemia, endothelial dysfunction

## Abstract

Thromboembolic events, such as acute limb ischemia, were reported worldwide in patients with COVID-19, suggesting that SARS-CoV-2 infection acts like a redoubtable prothrombotic factor in these patients. The aim of the study was to summarize the histopathological changes found in the arterial wall, intraarterial thrombus, and adjacent skeletal muscles. Considering the lack of evidence from in vivo studies, we performed observational histological research of peripheral arterial damage in patients with acute limb ischemia and SARS-CoV-2 infection. We investigated 22 patients with acute limb ischemia and SARS and harvested histopathological samples from those who agreed to this procedure. We performed histologic tissue harvesting during the revascularization procedure from the thrombosed area of the common femoral artery. Morphologic analysis was made on the hematoxylin-eosin (HE) stain. Special stains were also used—Elastica van Gieson (EvG) and Alcian Blue—Periodic Acid—Schiff (AB-PAS) and primary antibodies—CD45 and CD61. Our patients had significant risk factors for thrombus formation, since all of them had arterial hypertension, 81% had dyslipidemia, 73% were obese, 63% suffered from diabetes mellitus, and 45% were active smokers. The histological findings using immunohistochemistry (CD45 and CD68 reactions) or special and usual stains underlined the mechanism for ischemia production in SARS-CoV-2 patients. The main histological findings in our study were endothelial destruction and inflammation that were found in all analyzed structures.

## 1. Introduction

Since the pandemic outbreak in December 2019 in Wuhan, China, more than 240 million people have been infected with the SARS-CoV-2 virus, and 5 million have died. The pandemic affected more than 1.5 million people in Romania and killed almost 50,000 people [1,2,3,4]. As a respiratory virus, SARS-CoV-2 penetrates the human body and primarily infects the lungs. COVID-19 is often associated with fever, sore throat, dry cough, and shortness of breath [5]; nevertheless, several atypical instances and numerous asymptomatic individuals are being reported, which are more crucial to detect the restriction of viral transmission.

Although SARS-CoV-2 primarily affects the upper respiratory tract, unexpectedly, autopsies of COVID-19 patients showed thrombus formation in the lungs, heart, liver, and kidneys, and limbs, as well as blood clots causing strokes and heart attacks [6]. One of the unique features in SARS-CoV-2 infection are the vascular changes associated with the disease—formation of fibrin thrombi, diffuse intravascular coagulation, and large vessel thrombosis, either arterial or venous. Peripheral arterial vessels can also be affected, leading to acute limb ischemia of either inferior or superior limbs [7]. Over a third of COVID-19 patients with a severe form of the disease have been documented to have significantly altered plasma clotting values, such as high D-dimer values [8]. The formation of these inexplicable clots, which results in coagulation irregularities and thrombosis, is a legitimate cause for concern to the scientific world and, therefore, must be addressed.

Several studies describe endothelial dysfunction as the underlying mechanism of COVID-19 disease. This aspect that produces a series of inflammatory events at the local level can be the cornerstone for the future treatment of this disease [9]. On the other hand, all histological data resulted from a few autopsies performed on COVID-19 patients and showed small vessel thrombosis [10].

The study aimed to underline the histopathological aspects of arterial walls, adjacent skeletal muscles, and intraarterial thrombus in vivo from patients with SARS-CoV-2 infection and acute limb ischemia without any embolic causes.

## 2. Materials and Methods

### 2.1. Study Design

Patients with acute inferior limb ischemia (ALI) and SARS-CoV-2 infection were included in the present study. The clinical status of the patients was defined using the Rutherford classification system [11]. SARS-CoV-2 infection was diagnosed with a RT-PCR test for all patients, and they all underwent chest-computed tomography that represents the standard of care in our hospital. All patients underwent preoperative blood tests that consisted of blood count, creatin-phosphokinase (CK), urea, creatinine, LDH, CRP, D-dimers, etc. A complete history was taken from all the patients. We performed computed tomography angiography (CT-Angio) for all patients to assess the extension of the arterial lesions, according to the standard of care for acute limb ischemia [12]. The surgical treatment was individualized for every patient considering the aspect and extent of peripheral lesions. All patients were isolated, and all procedures were performed by observing the universal caution regarding the SARS-CoV-2 infection, avoiding cross-contamination, and reducing the risk of unnecessary viral spread. All patients were treated surgically or through endovascular techniques.

### 2.2. Histological Methods

We performed histologic tissue harvesting (arterial wall, interstitial muscle, and intraarterial clots) during the revascularization procedures from the common femoral artery from the thrombosed area, trying to underline the vessel wall abnormalities in SARS-CoV-2 infection. Specimen sectioning was followed by primary processing of the samples consisting of 10% formaldehyde fixation for 24 h, paraffin inclusion according to the standard technique (washing, dehydration, clearing, inclusion), and section cutting at 3–4 µm. Morphologic analysis was made on hematoxylin-eosin (HE)-stained sections.

For the immunohistochemistry (IHC) and special stains, additional sections 3–4 µm thick were cut from the selected paraffin blocks. For the IHC reactions, the additional sections were placed on Super Frost Ultra Plus slides. The following primary antibodies were used: CD45 [clone 2B11 + PD7/26, DAKO, ready to use (RTU)] and CD68 (clone PG-M1, Dako, RTU). Antigen retrieval was performed by Heat Induced Epitope Retrieval (HIER) in target retrieval solution pH6 for CD45 and pH9 for CD68, for 20 min at 98 °C. After incubation with primary antibodies (for 15–30 min), we used a horseradish peroxidase (HRP)—polymer detection system (Novolink)—for 30 min, followed by the visualization of the reaction with 3,3′-diaminobenzidine (DAB) chromogen (for 5 min) and then counterstained with hematoxylin (for 3 min).

For the Elastica van Gieson (EvG) stain, additional sections were deparaffinized in a conventional manner and rehydrated in a descending alcohol series. According to standard procedures, a routine Elastica van Gieson staining kit (Merck, Catalog No. 1.15974.0002) was used. Finally, sections were dehydrated (ascending alcohol series), cleared with xylene, and mounted.

For the Alcian Blue—Periodic Acid Schiff (AB-PAS) stain, additional sections were deparaffinized in a conventional manner and hydrated with distilled water. AB-PAS (Bio-Optica) staining was performed according to standard procedures. Finally, sections were dehydrated (ascending alcohol series), cleared with xylene, and mounted.

### 2.3. Ethics

All patients signed an informed consent for the treatment and for the use of their clinical files under proper anonymization. The study has the agreement of the Ethics Committee of the “Pius Brînzeu” Clinical County Emergency Hospital, under the EU GCP Directives, International Conference of Harmonization of Technical Requirements for Registration of Pharmaceuticals for Human Use (ICH) and Declaration of Helsinki (No. 189/04.05.2020).

### 2.4. Statistical Analysis

Data were analysed with MedCalc Statistical Software version 19 (MedCalc Software bvba, Ostend, Belgium). Data are presented as mean and standard deviation, median and interquartile range (IQR), total count and frequency.

## 3. Results

Twenty-two patients aged 43–86 (mean age 64.91 ± 9.57 years) were admitted to the Vascular Surgery Department of the “Pius Brinzeu” Emergency County Hospital Timisoara with acute inferior limb ischemia and SARS-CoV-2 infection. None of the patients presented with atrial fibrillation or signs of embolic risk factors (transthoracic ultrasound did not reveal any cardiac thrombus). There were 15 males (68.18%) and seven females (31.82%). Patients’ demographic data are presented in Table 1. In Table 2 we show the main laboratory findings in our sample. Fourteen patients (63.64%) had no specific symptoms for COVID-19 infection, while the other 8 patients had mild COVID-19 symptoms, such as dyspnea and loss of smell and taste. All patients were known to have arterial hypertension, while 18 (81.8%) had dyslipidemia, and 14 (63.6%) suffered from diabetes mellitus. Another prominent characteristic of this cohort was class I obesity, patients having an average BMI of 31 kg/m^2^. It is essential to mention that 10 (45.4%) patients were smokers, and 19 (86.3%) followed antiplatelet treatment at home. Among the study participants, 15 (68.18%) were classified as Rutherford IIA and 7 (31.815) were Rutherford IIB.

Open surgery was performed under local or loco-regional anesthesia and consisted of Fogarty^®^ balloon embolectomy (Le Maître, Burlington, MA, USA), followed by histopathological harvesting. All the histological samples were obtained from the common femoral artery. The diameter of the arteries varied between 5 and 7 mm. The majority of arterial walls from the biopsied arteries were elastic, with little or no atheromatous plaque formation at the level of the approached site. The fresh red clot thrombotic material was easily removed and had a medium length of 20–30 cm. The muscle fragments were harvested from the sartorius muscle of the inguinal region.

**Figure 1:**
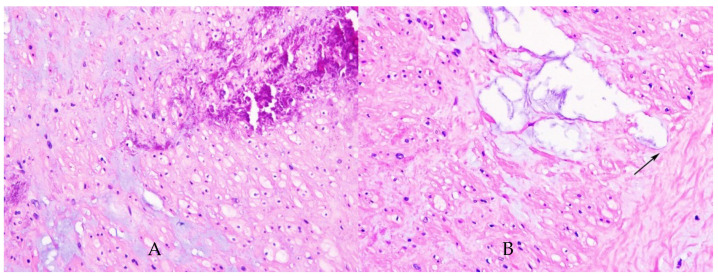
Haematoxylin-eosin coloration. (**A**) High-power view of the artery with medial calcification and myxoid degeneration. HE, ×400. (**B**) High-power view of the medial myxoid degeneration. Notice the cystic spaces which encounter external elastic lamina (arrow); HE, ×400.

**Figure 2:**
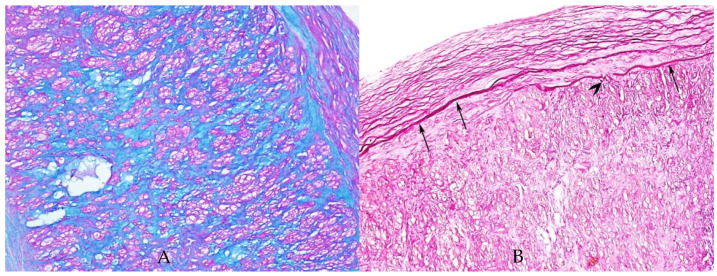
(**A**) AB-PAS stain highlights acid mucins (blue stain) within the entire wall of the artery; ×200. (**B**) Elastica van Gieson stain highlights the internal elastic lamina (arrows) which is focally interrupted (arrowhead), ×100.

We also observed on our sample endothelial injury with the destruction of the internal elastic lamina (AB-PAS stain) and with cystic spaces that encounter external elastic lamina (HE stain) (Figure 1 and Figure 2). The endothelial destruction, also known as endothelitis [13], is followed by a series of events, that is, platelet activation, aggregation, and adhesion, and finally, the formation of clots inside the blood vessels.

**Figure 3:**
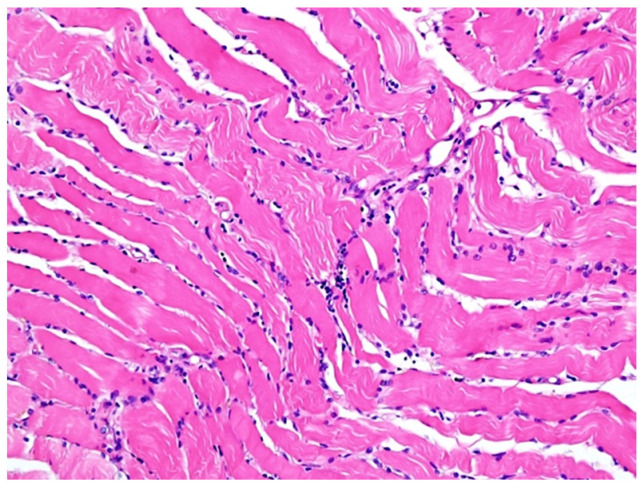
Haematoxylin-eosin coloration. Microscopic features of skeletal muscle inflammation; interstitial aggregates of lymphocytes and macrophages can be noticed; HE, ×200.

**Figure 4:**
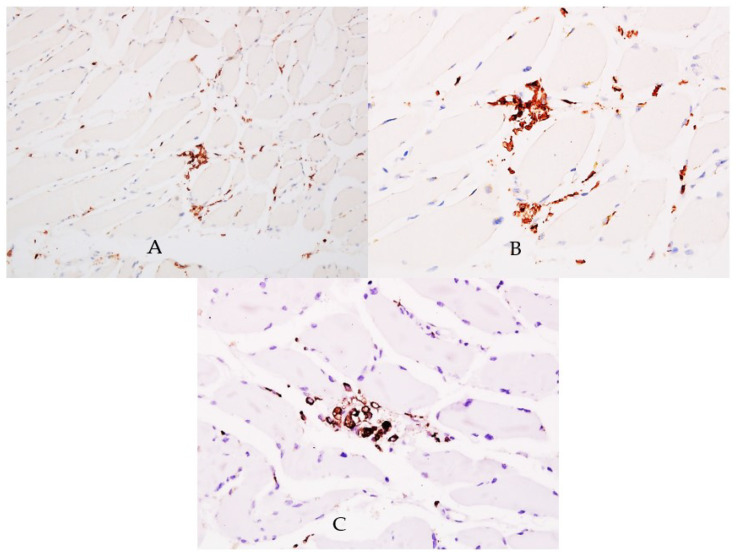
CD45 reaction. (**A**) Lymphocytes within muscle interstitium are highlighted by CD45 positive reaction, ×200. (**B**) High-power view of the interstitial lymphocytic infiltrate, positive for CD45, ×400. (**C**) Aggregates of macrophages positive for CD68 were noticed within the skeletal muscle interstitium, ×400.

Biopsy from striate muscle showed interstitial aggregates of lymphocytes and macrophages and inflammation (Figure 3). Myocytes were apparently normal and there were no viral particles. Although viral particles were absent, other signs of inflammation like lymphocytes with positive CD45 reaction (Figure 4A,B) and aggregates of macrophages positive for CD68 reaction (Figure 4C), indicating indirect injury to the cells by the SARS-CoV-2 were found.

**Figure 5:**
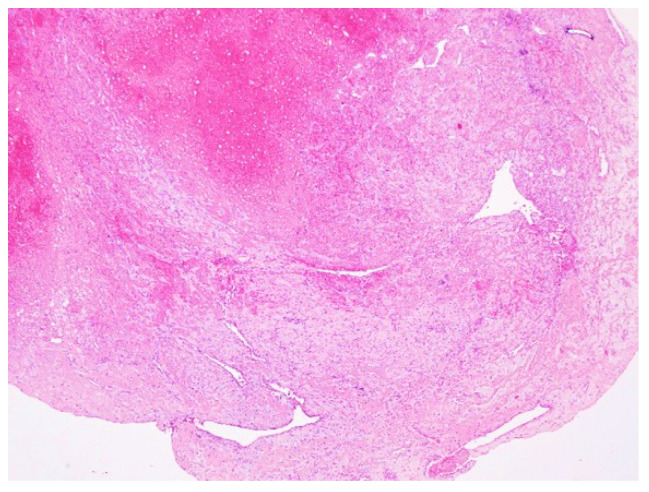
Haematoxylin-eosin coloration. Well-organized thrombus showing recanalization with vascular channels lined by endothelium; HE, ×40.

After analyzing the thrombus, we found vascular channels lined by the endothelium (Figure 5), also a suggestion of an inflammatory reaction.

## 4. Discussion

The main factors involved in the pathogeny of SARS-CoV-2 infection are hypercoagulability, hypofibrinolysis, and platelet hyperreactivity [9]. Several studies [10] underline that endothelial injury and disruption are the first occurrences in patients with COVID-19. The ACE-2 (angiotensin-converting enzyme 2) receptor is physiologically expressed at the surface of endothelial cells and results in direct viral infection [14].

All patients suffering from SARS-CoV-2 infection have a high prevalence of thrombotic events, considering many studies describing a high prevalence of venous thromboembolism. Klok et al. reported 31% of thrombotic events in a series of 184 critically patients [15], and Helms reported 16.7% of pulmonary embolism and 2% vein thrombosis [16].

In patients that did not have evidence of thrombosis at the macrovascular level, there were findings at the microvascular one, that is, small thrombi in pulmonary arterioles [17], superficial dermal vessels, glomerular capillary [18,19], and complete luminal thrombosis in small and medium-sized arteries [20]. Unfortunately, only a few autopsies were performed because of several limitations and the high level of infectiousness of the virus.

An important study on 26 patients who died from COVID-19 pneumonia came to underline the vascular lesions, like endothelitis and thrombotic angiopathy. This study also underlines the fact that we did not yet know whether these lesions were related strictly to a viral presence or were due to the accumulation of other factors [21].

The main histological findings in our study were endothelial alteration and inflammation that were found in all analyzed structures. The endothelium is the main feature that maintains vascular homeostasis, and its alteration results in altered vascular equilibrium, organ ischemia, inflammation, edema, and a procoagulant state [22].

In our study, the endothelial damage was associated with lymphocytic infiltrate and the presence of an inflammatory reaction, facts demonstrated also by other studies in the lungs of patients that died from COVID-19 [23].

A study conducted by Roncati et al. underlined the phenomenon of ”leukocytoclastic vasculitis” that consists in the tunica media and adventitia infilitration with neuthrophils, activation of polyclonal immune complexes and of the C3 fraction of the complement, leading to an increased risk of thrombosis. Furthermore, this sequences can be amplified by the presence of antiphospholipid antibodies generating the hypercoagulant status or ”immunothrombosis”. In their paper, the authors also mention that the medium-sized arteries can develop complete obstruction, and large vessels like aorta can develop a mural thrombus [24].

One important piece of information about thrombosis in patients with COVID-19 is related to the association between symptoms and arterial or venous thrombosis. Several articles stated that patients with arterial thrombosis usually have less severe symptoms, while those with venous thrombosis have more severe symptoms [25]. There were observed other differences as well, this time regarding the higher platelet activation-related with venous thrombosis (higher platelet count, but not platelet volume). In our study, we could not find this association, but there were patients with high values of platelets [26].

Other studies also showed the levels of cardiovascular system modifications, such as acute necrosis, presence of inflammatory cells and apoptotic bodies, and foci of lymphocytic inflammation [27,28,29,30].

On the CD45 reaction, lymphocytes within muscle interstitium were found, while another study showed plump endothelial cells in lymphoplasmacytic infiltrate surrounded venules without intraluminal thrombi [31]. Our study has several limitations: the small sample of patients, single-center experience, and the fact that we could not technically isolate the virus from the paraffin-embedded tissues.

## 5. Conclusions

Although our study was observational, it contributes to the identification of microscopic aspects in patients with acute limb ischemia and SARS-CoV-2 infection.

Further studies are necessary to demonstrate the links between infection and thrombotic events from in vivo samples.

## Figures and Tables

**Table 1:** Demographic data and patients’ characteristics.

Variable, n (%) *	Data
**General Characteristics**	
Age, years (mean ± SD)	64.91 ± 9.57
Sex, male	15 (68.18)
BMI, kg/m^2^ (mean ± SD)	31.63 ± 6.47
Smoking	10 (45.45%)
Ischemia time, hours (median, IQR)	18.59 (5–34)
Preoperative antiplatelet treatment	19 (86.36)
**Comorbidities**	
Heart Failure	8 (36.36)
Obesity	16 (72.72)
Diabetes mellitus	14 (63.64)
Dyslipidemia	18 (85.71)
Arterial hypertension	22 (100)
Chronic obstructive pulmonary disease	4 (18.18)
Cerebrovascular disease	4 (18.18)
Malignancy	2 (9.09)
**Rutherford classification**	
IIA	15 (68.18)
IIB	7 (31.81)

* Unless specified differently; SD—Standard Deviation; IQR—Interquartile Range; BMI—Body Mass Index.

**Table 2:** Preoperative laboratory findings in our study.

Characteristic	Patients’ Values	Range Values
Leukocyte count (no.×10^3^/L), median [IQR]	8.35 [5.34–14.28]	4–9.5
Neutrophils (%), mean ± SD	62.28 ± 12.42	45–70%
Erythrocyte count (no.×10^3^/L), median [IQR]	3.64 [3.45–4.24]	4–5.5
Monocyte, median [IQR]	7.34 [2.89–8.28]	3.5–9%
Lymphocyte (no.×10), median [IQR]	1.33 [1.09–1.77]	0.8–3.8
Haemoglobin level (g/dL), median [IQR]	10.70 [10.31–11.40]	11.5–15
Haematocrit (%), mean ± SD	34.08 ± 3.47	35–46
Platelet count, mean ± SD	275,545 ± 82,299	150–400
LDH, median [IQR]	278 [161.3–346.5]	120–246
Ferritin level (µg/L), mean ± SD	728.9 ± 158.5	20–290
CRP level (mg/L), mean ± SD	68.08 ± 23.67	0–10
aPTT (s), median [IQR]	29.4 [24.4–35.41]	25.1–36.5
Quick time (s), median [IQR]	14.67 [12.68–15.61]	9.4–12.5
INR, mean ± SD	1.27 ± 0.18	0.8–1.07
VSH (mm/1 h), mean ± SD	82.41 ± 22.26	1–15
AST (U/L), median [IQR]	23.5 [18–28.25]	14–36
ALT (U/L), mean ± SD	23.91 ± 10.45	0–35
DDimers (ng/mL) mean ± SD	957 ± 518.6	0–243
Urea (mg/dL), median [IQR]	30 [23–45]	15–36
Creatinine (mg/dL), median [IQR]	0.89 [0.7–1.42]	0.7–1.2
Fibrinogen (mg/dL), mean ± SD	668 ± 168.3	200–393
CK (U/L), median [IQR]	115 [43.75–508.8]	30–170

## Data Availability

Data available on request.
